# Supplementary material for: A single-cell atlas enables mapping of homeostatic cellular shifts in the adult human breast
Source: Nat Genet. 2024 Mar 28;56(4):652–62. doi: 10.1038/s41588-024-01688-9 (PMC11018528; doi:10.1038/s41588-024-01688-9)
Supplement: Supplementary file 1 — Supplementary Figs. 1–15. [file 41588_2024_1688_MOESM1_ESM.pdf]

nature genetics

Article https://doi.org/10.1038/s41588-024-01688-9

A single-cell atlas enables mapping of homeostatic cellular shifts in the adult human breast

In the format provided by the authors and unedited

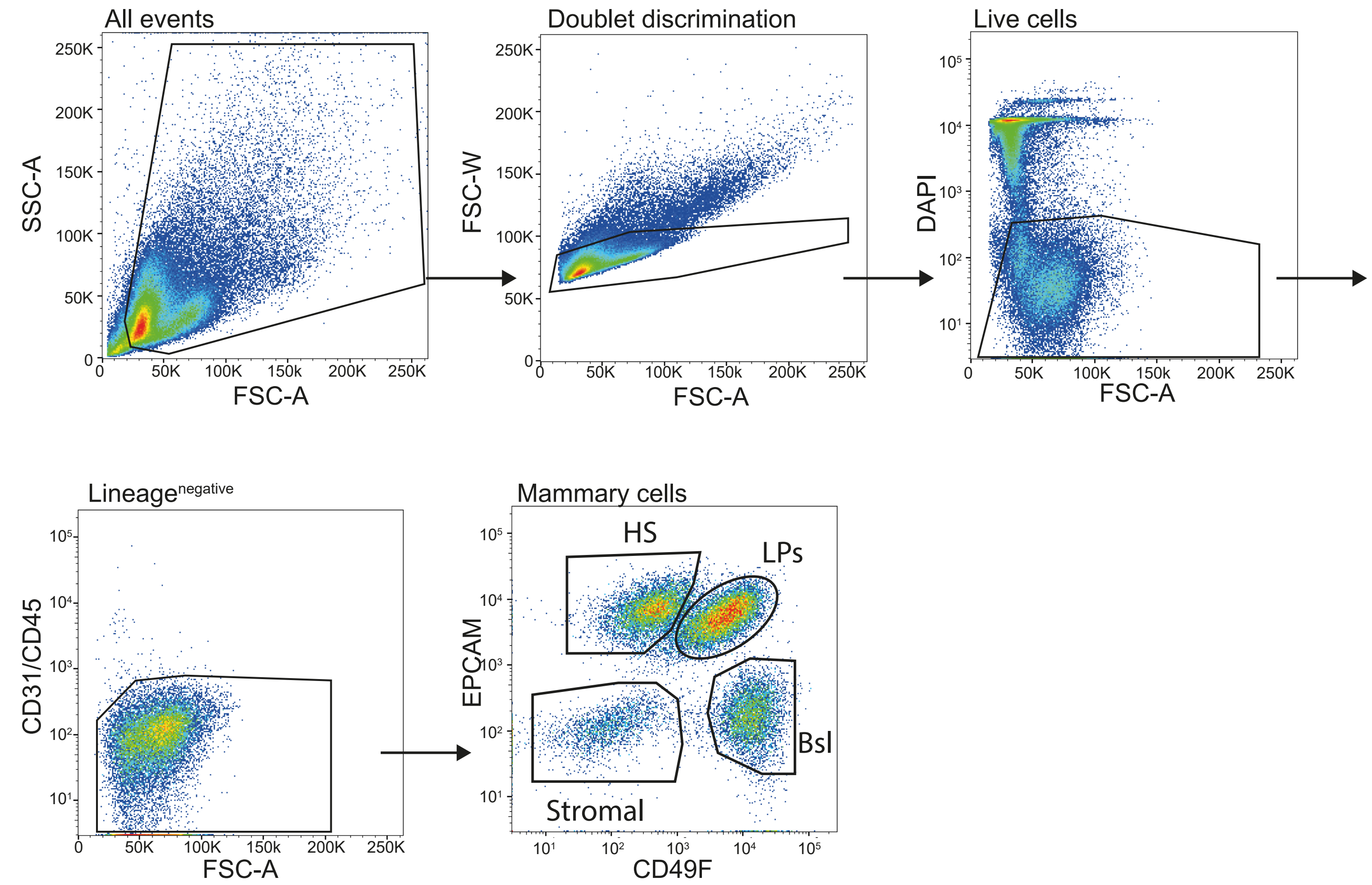

Supplementary Figure 1. Gating strategy used for human samples on the FACS Aria Fusion sorter

Representative plots showing the gating strategy used to select live, lineage negative, single luminal progenitor cells based on EPCAM and CD49f staining of single cell preparations. FSC-W: forward scatter width, FSC-A: forward scatter area, SSC-A: side scatter area. The arrows indicate sequential gating. This gating strategy was used for all samples.

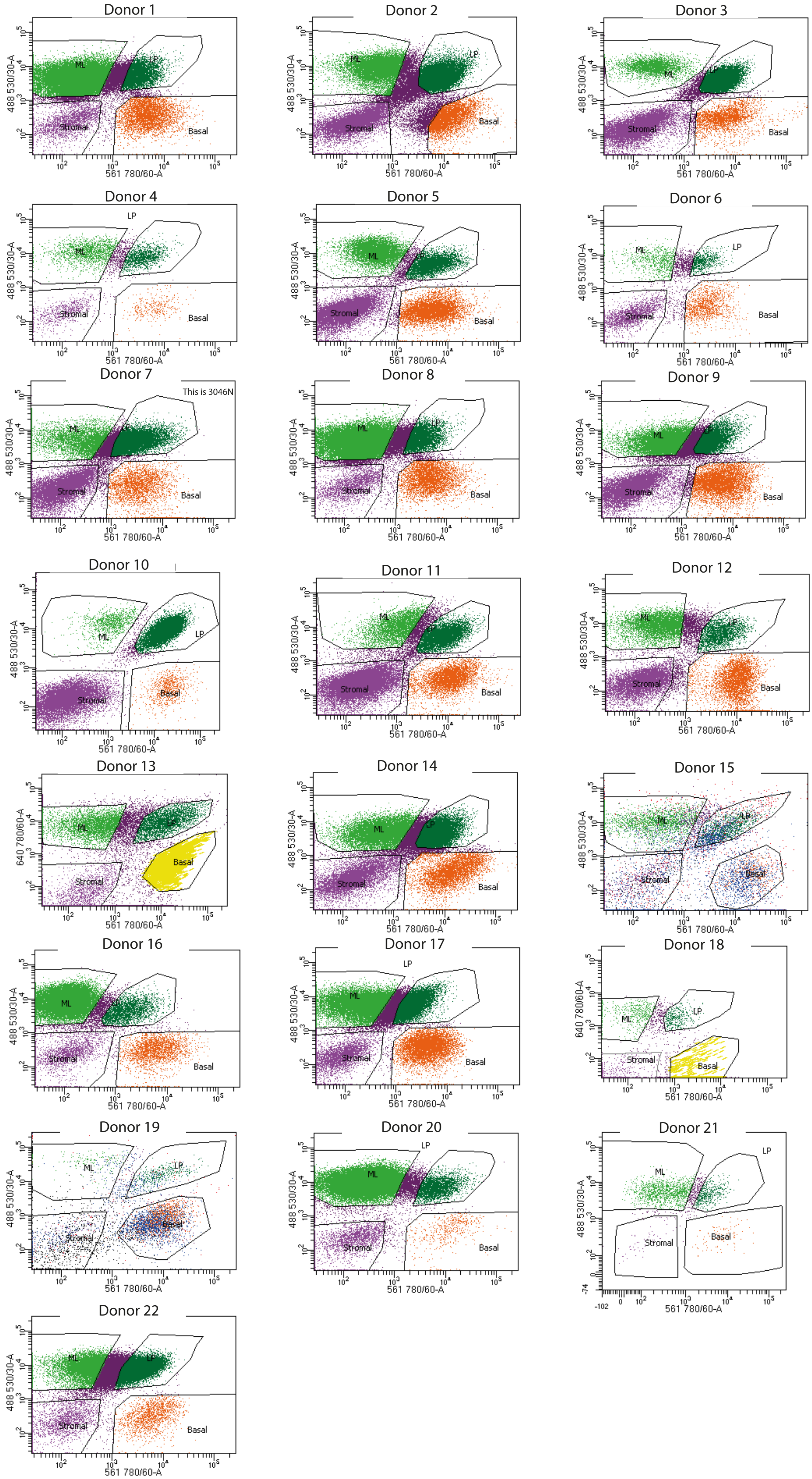

Supplementary Figure 2. Individual samples FACS plots for mammoplasty donors

FACS plots showing EPCAM (shown as 488 530/30-A) and CD49f (shown as 561 780/60-A) expression on live, lineage negative, single cells for individual mammoplasties samples. ML indicates luminal hormone sensing population, LP indicates the luminal adaptive secretory precursors and basal indicate the basal-myoepithelial cells. Donor IDs are indicated on each plot.

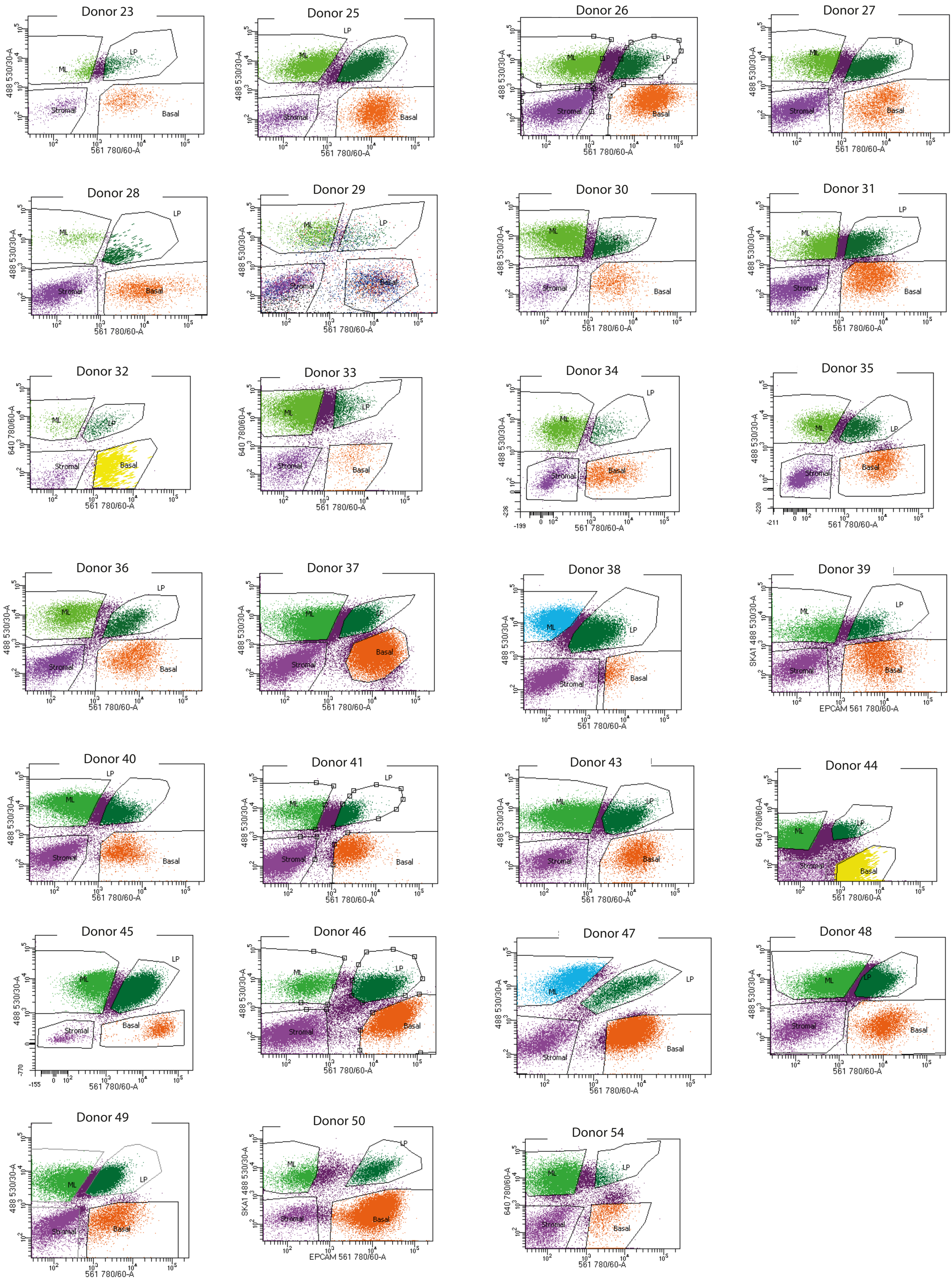

Supplementary Figure 3. Individual samples FACS plots for mastectomy and contralateral donors

FACS plots showing EPCAM (shown as 488 530/30-A) and CD49f (shown as 561 780/60-A) expression on live, lineage negative, single cells for individual mastectomies and contralateral samples. ML indicates luminal hormone sensing population, LP indicates the luminal adaptive secretory precursors and basal indicate the basal-myoepithelial cells. Donor IDs are indicated on each plot.

Supplementary Figure 4 **Reed. et al**

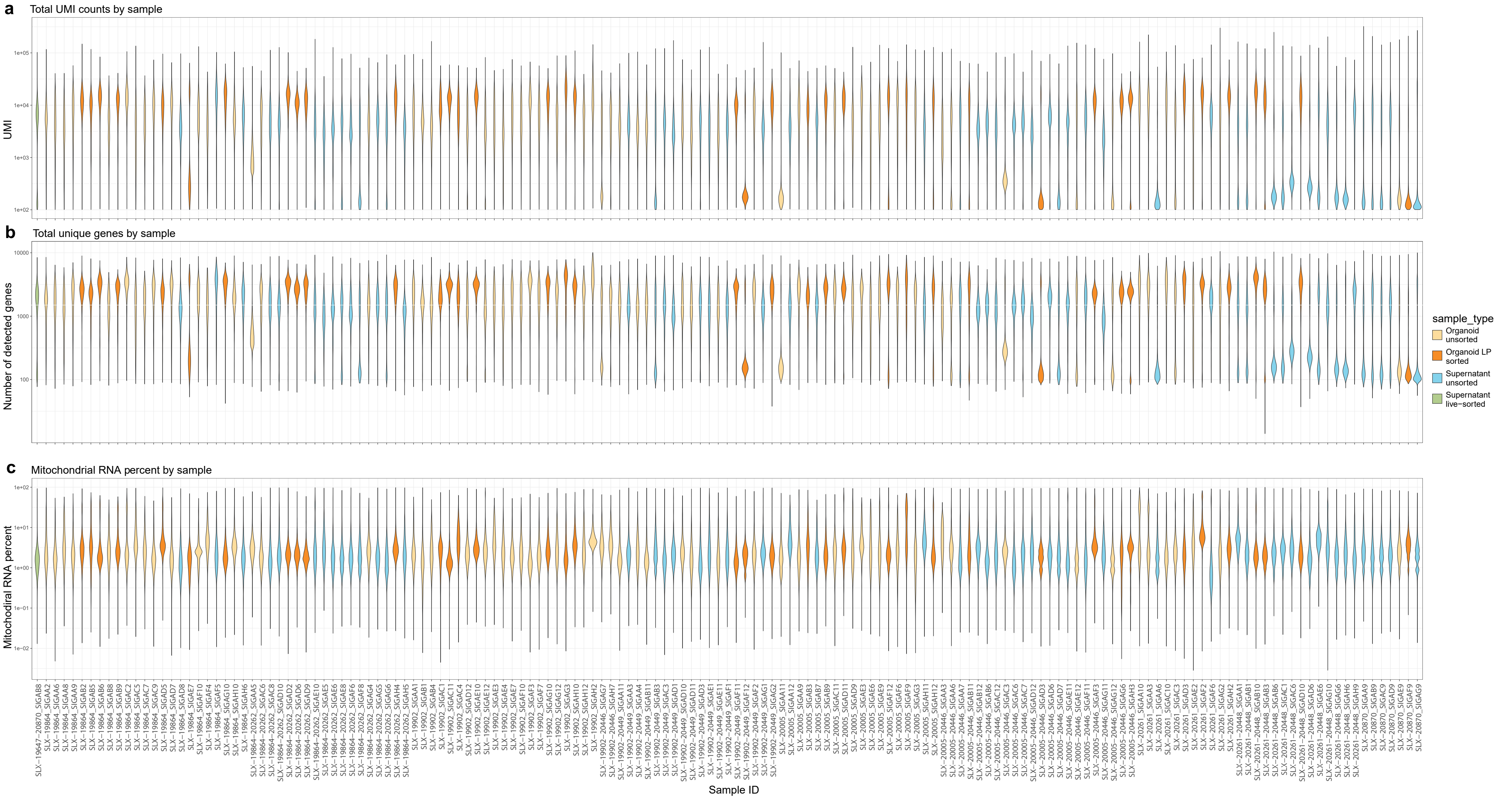

Supplementary Figure 4. Pre-filter sample quality control metrics

(a) A collection of violin plots for the quality control unique molecular identifiers (UMI) counts per 10X lane (sample_id) prior to the application of relevant cutoffs (methods).

(b) A collection of violin plots for the quality control (unique) gene counts per 10X lane (sample_id) prior to the relevant cutoffs (methods).

(c) A collection of violin plots for the quality control mitochondrial RNA proportions per 10X lane (sample_id) prior to the relevant cutoffs (methods).

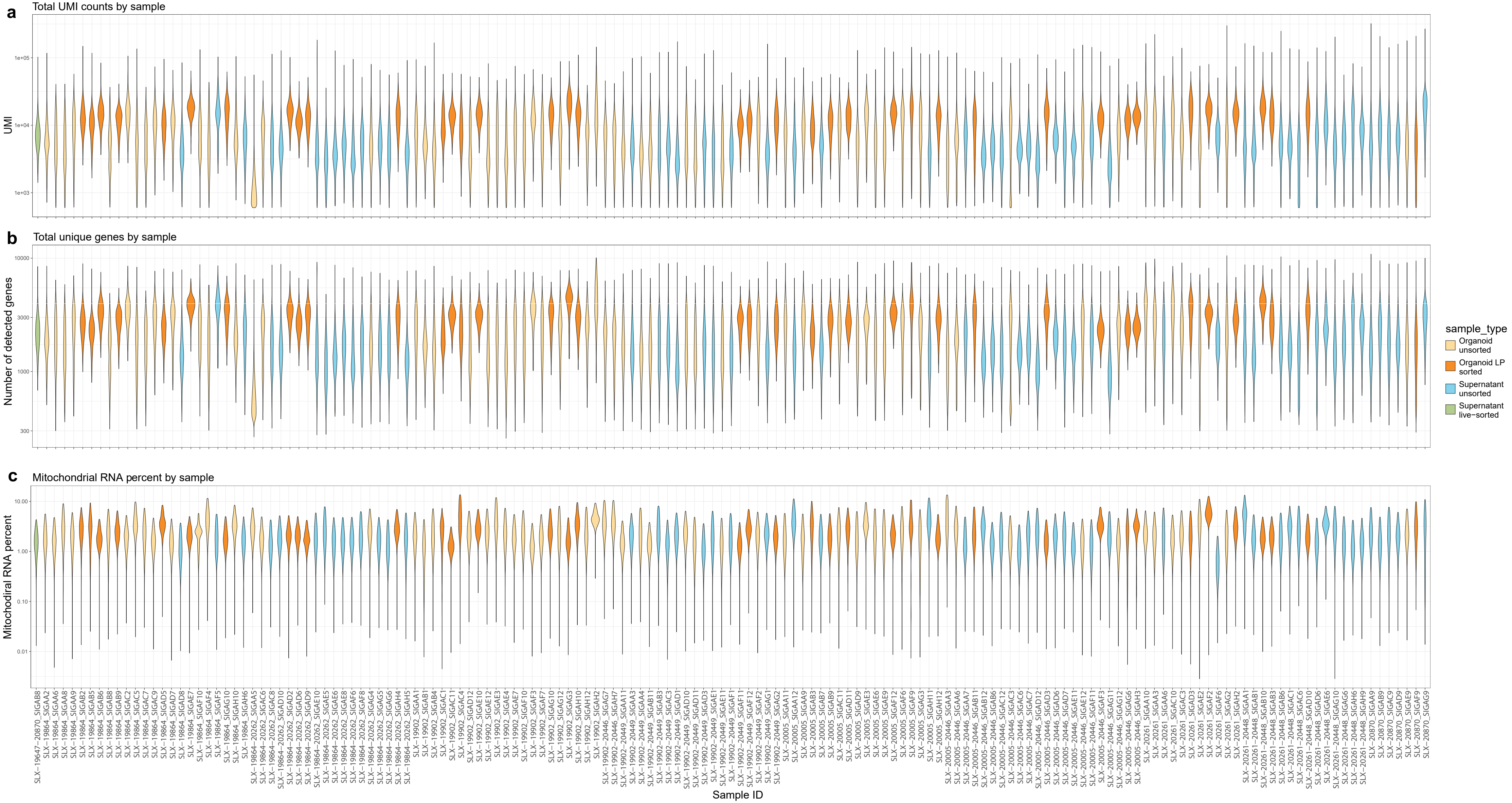

Supplementary Figure 5. Post-filter sample quality control metrics

(a-c) Same plots as Supplementary Figure 5 but for the cleaned (post-filter) cells passing the necessary quality control thresholds (see methods).

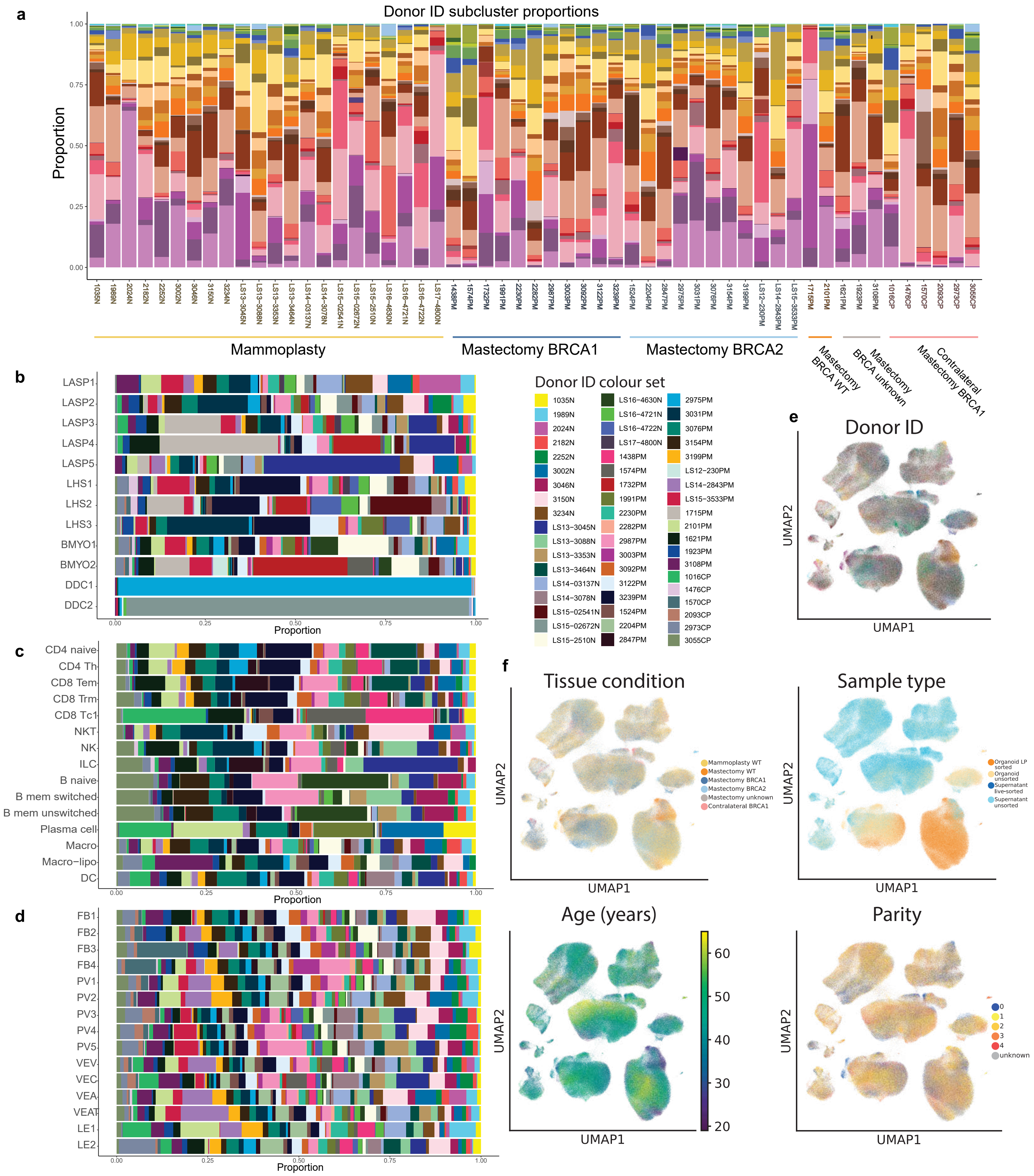

Supplementary Figure 6. Subcluster donor and demographic heterogeneity

(a) Stacked bar plots displaying the proportional cellular composition of each donor normalised on a per donor basis.

(b-d) Bar plots displaying the proportions of donor contribution per cell type subcluster for the epithelial (top), stromal (middle) and immune (bottom) compartments respectively.

(e-f) Uniform manifold approximation and projection (UMAP) plots of all cells coloured by their respective donor, tissue condition (collection surgery performed and BRCA status), age, parity and sample type (see Figure 1 for types of 'enriched cell' sample types).

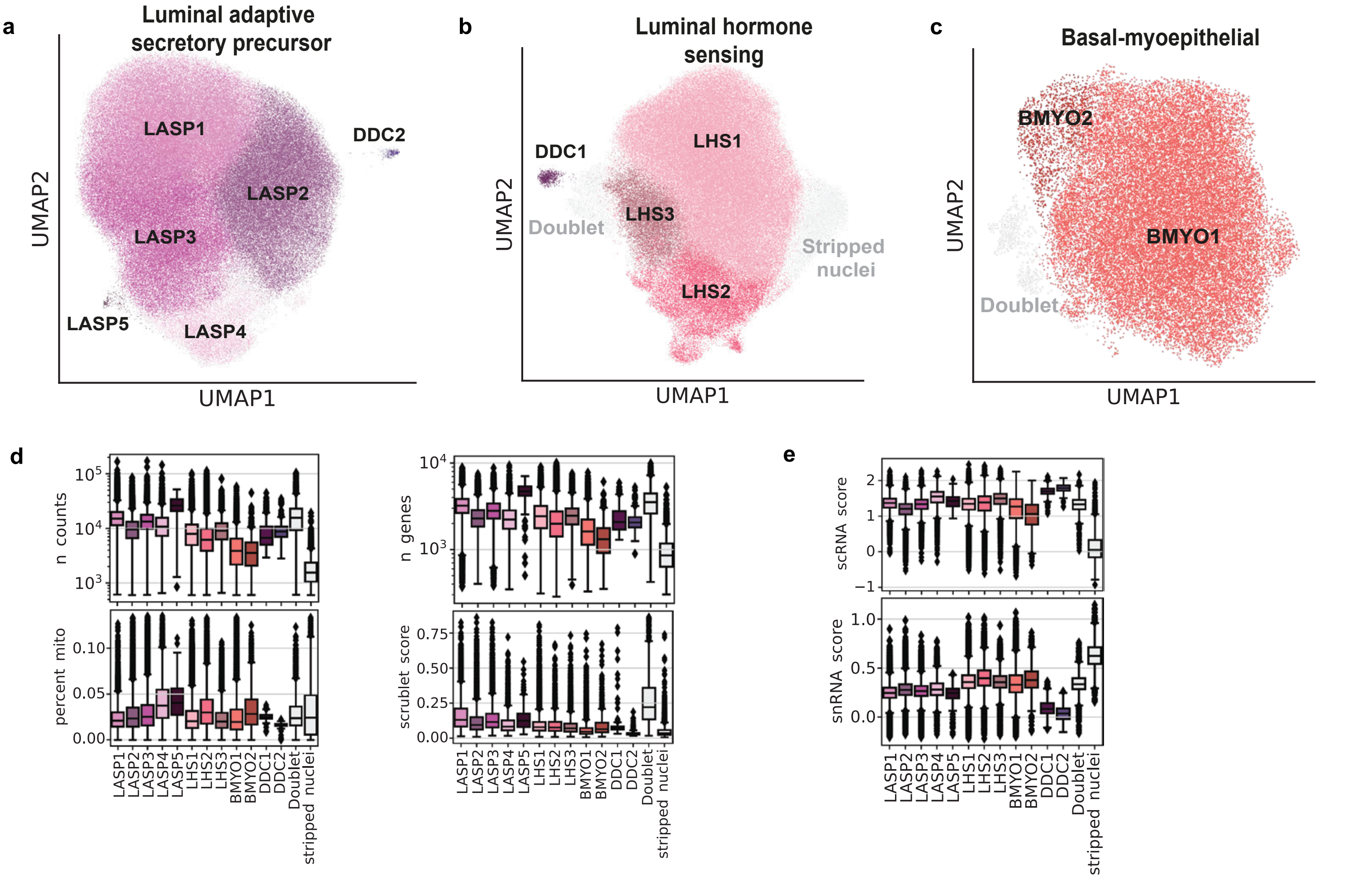

Supplementary Figure 7. Epithelial cell type and subcluster annotation

(a-c) Cell type specific uniform manifold approximation and projections (UMAPs) for the luminal adaptive secretory precursor, luminal hormone sensing and basal-myoepithelial cell types used to identify subclusters.

(d) Boxplots showing the distribution of Scrublet (doublet) scores, unique molecular identifier (UMI) counts, unique gene counts and mitochondrial RNA percentages across the epithelial subclusters (all samples; n=55). Combined with marker gene expression these were used to identify any additional doublet clusters annotated in (a-c). The boxplot centers show median values while the minima / maxima show the 25th /75th percentiles respectively and whiskers extend to the most extreme datapoint within 1.5 x IQR (inter-quartile range) of the outer hinge of the boxplot. Outliers are then displayed independently.

(e) Single nucleus RNA (snRNA) and single cell RNA (scRNA) sequencing gene signature scores for each of the defined subclusters (all samples; n=55). The boxplot centers show median values while the minima / maxima show the 25th /75th percentiles respectively and whiskers extend to the most extreme datapoint within 1.5 x IQR (inter-quartile range) of the outer hinge of the boxplot. Outliers are then displayed independently.

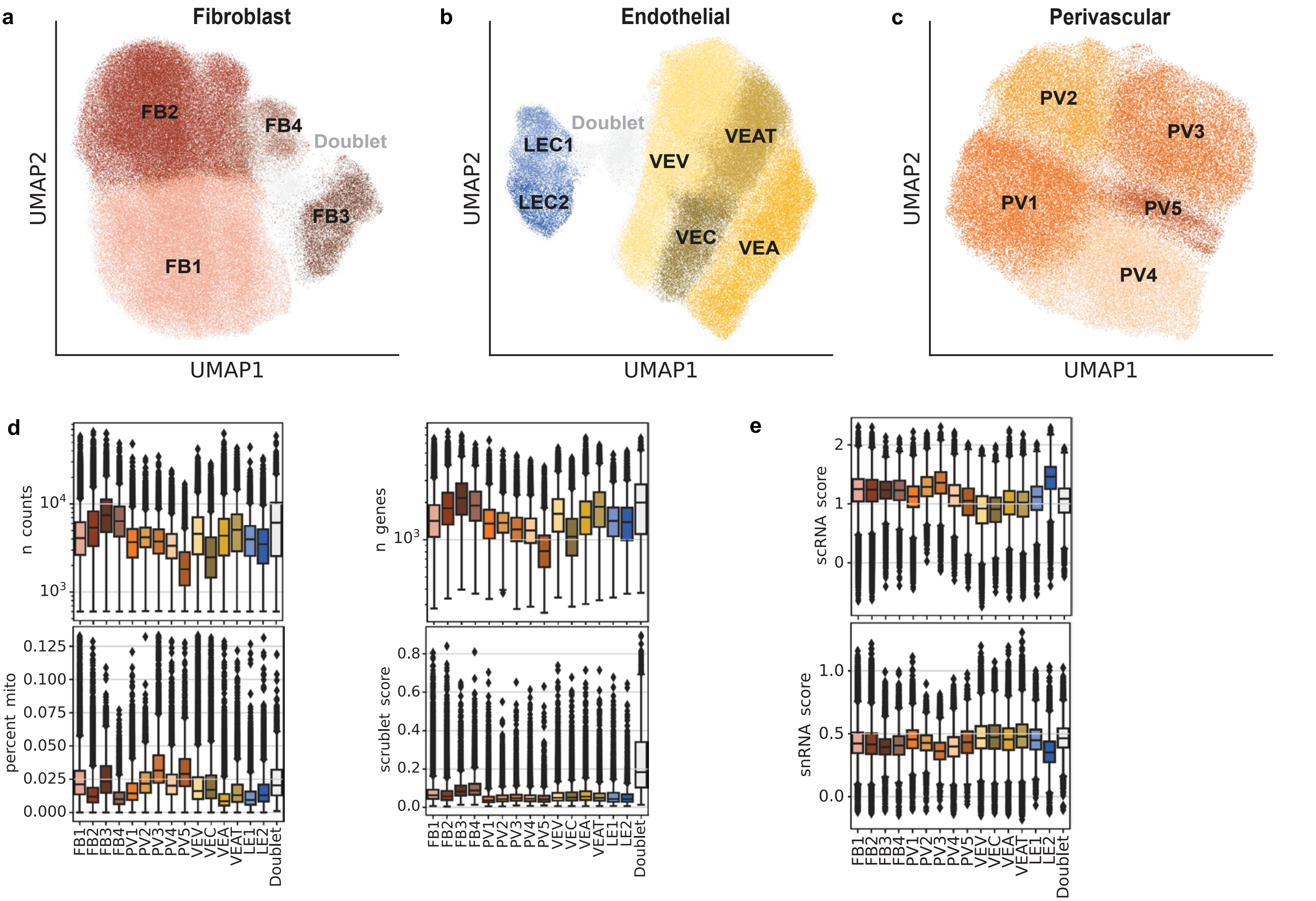

Supplementary Figure 8. Stromal cell type and subcluster annotation

(a-c) Cell type specific uniform manifold approximation and projections (UMAPs) for the fibroblast, endothelial and perivascular cell types used to identify subclusters.

(d) Boxplots showing the distribution of Scrublet (doublet) scores, unique molecular identifier (UMI) counts, unique gene counts and mitochondrial RNA percentages across the stromal subclusters (all samples; n=55). Combined with marker gene expression, these were used to identify additional doublet clusters annotated in (a-c). The boxplot centers show median values while the minima / maxima show the 25th /75th percentiles respectively and whiskers extend to the most extreme datapoint within 1.5 x IQR (inter-quartile range) of the outer hinge of the boxplot. Outliers are then displayed independently.

(e) Single nucleus RNA (snRNA) and single cell RNA (scRNA) sequencing gene signature scores for each of the defined subclusters (all samples; n=55). The boxplot centers show median values while the minima / maxima show the 25th /75th percentiles respectively and whiskers extend to the most extreme datapoint within 1.5 x IQR (inter-quartile range) of the outer hinge of the boxplot. Outliers are then displayed independently.

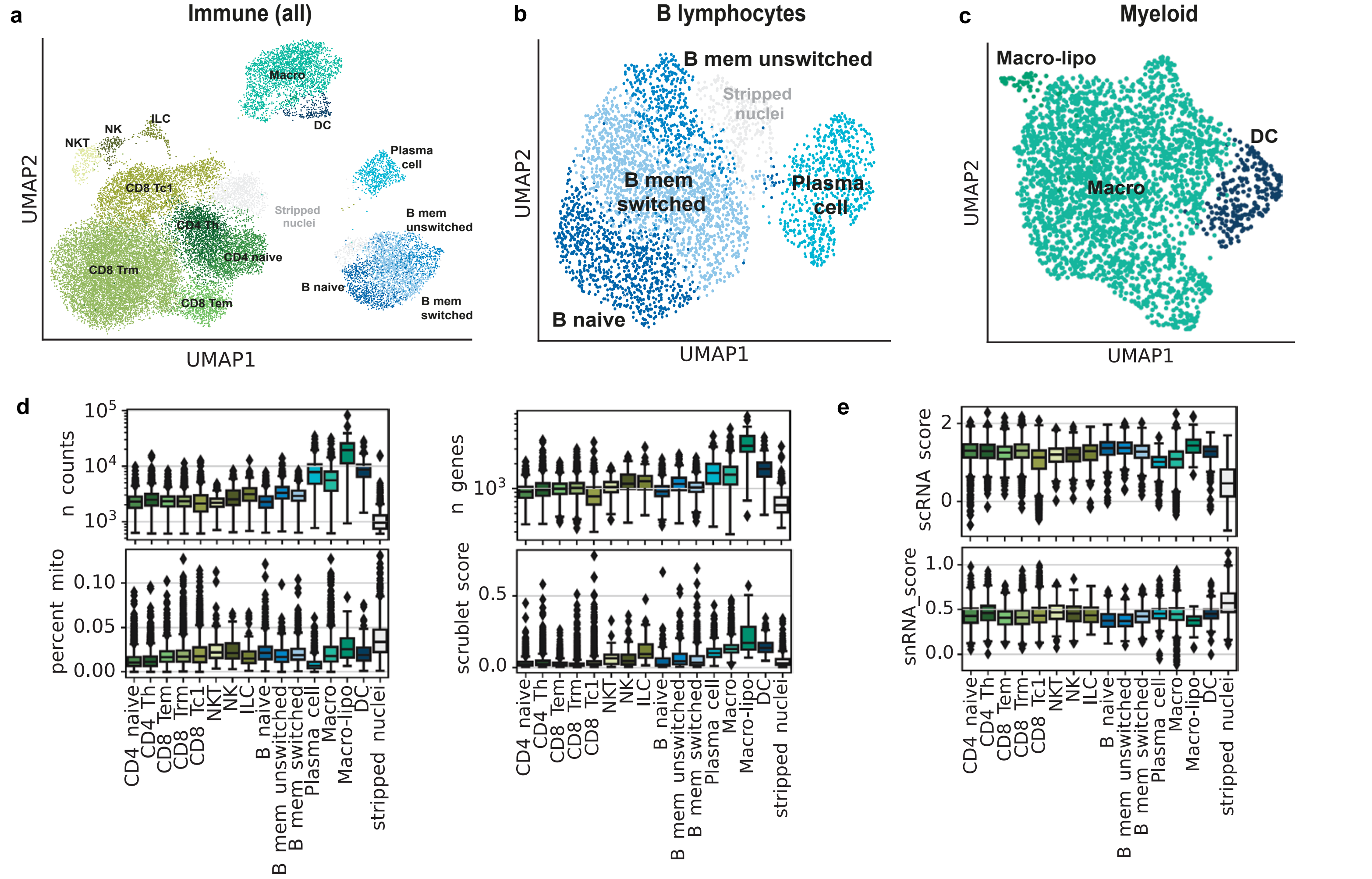

Supplementary Figure 9. Immune cell subcluster annotation

(a-c) Cell type specific uniform manifold approximation and projections (UMAP) for all immune cell types used to identify subclusters with additional subUMAPs for the B lymphocytes and myeloid cells.

(d) Boxplots showing the distribution of Scrublet (doublet) scores, unique molecular identifier (UMI) counts, unique gene counts and mitochondrial RNA percentages across the immune cell subclusters (all samples; n=55). Unlike the epithelial and stromal compartments, these show no doublet clusters identified here and that they were removed in earlier stages. The boxplot centers show median values while the minima / maxima show the 25th /75th percentiles respectively and whiskers extend to the most extreme datapoint within 1.5 x IQR (inter-quartile range) of the outer hinge of the boxplot. Outliers are then displayed independently.

(e) Single nucleus RNA (snRNA) and single cell RNA (scRNA) sequencing gene signature scores for each of the defined subclusters (all samples; n=55). The boxplot centers show median values while the minima / maxima show the 25th /75th percentiles respectively and whiskers extend to the most extreme datapoint within 1.5 x IQR (inter-quartile range) of the outer hinge of the boxplot. Outliers are then displayed independently.

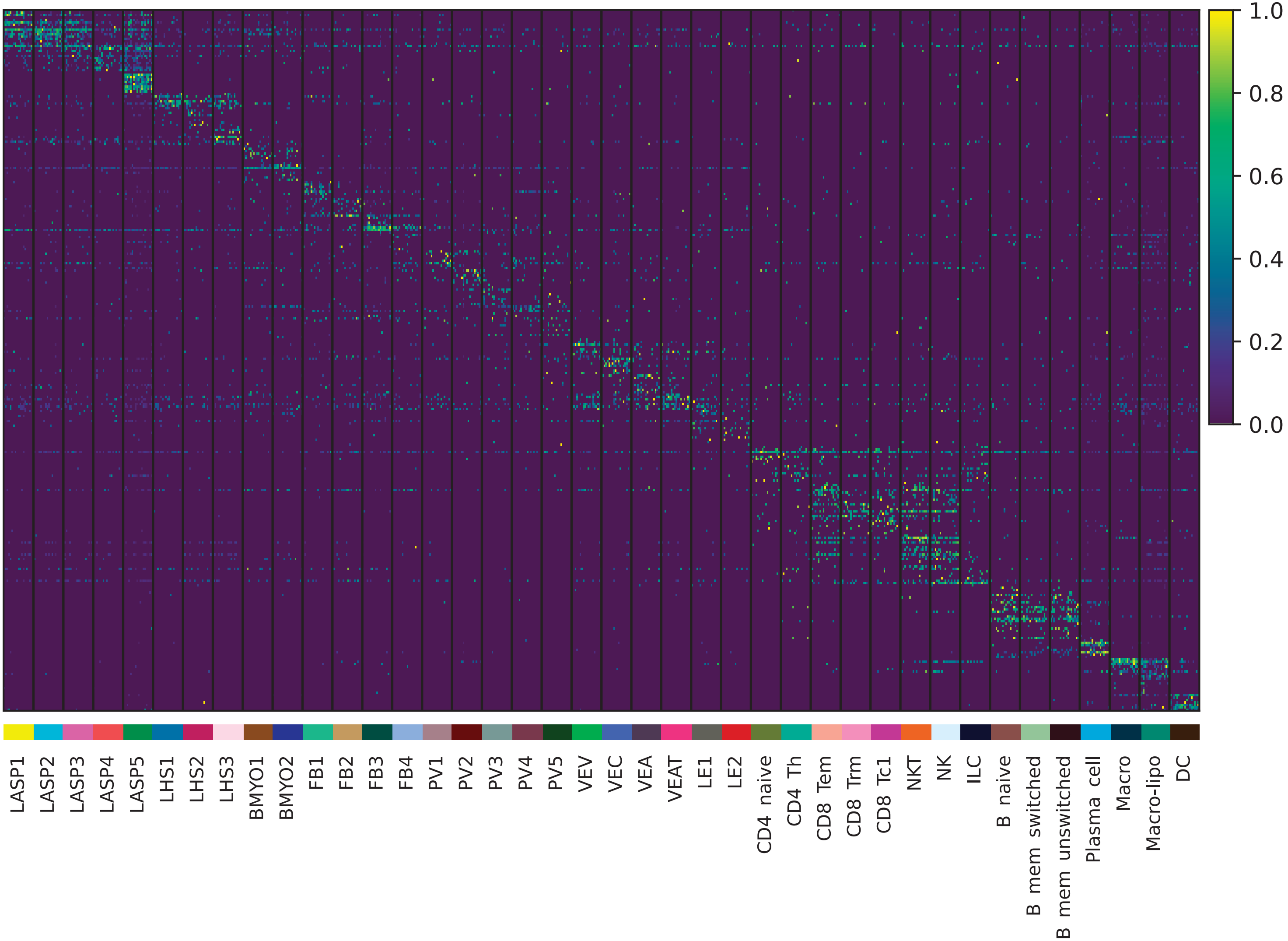

Supplementary Figure 10. Specificity of subcluster gene signatures
Heatmap showing the top 10 marker genes (rows) for each subcluster identified via several 1 vs all negative binomial tests for each (levlel2) subcluster (columns; subsetted to 100 cells per subcluster). Full lists of the top 100 genes per subcluster are detailed in Supplementary Table 4. The heatmap was made using scanpy.pl.heatmap() with parameters "standard_scale='var'" and "layer='logcounts'".

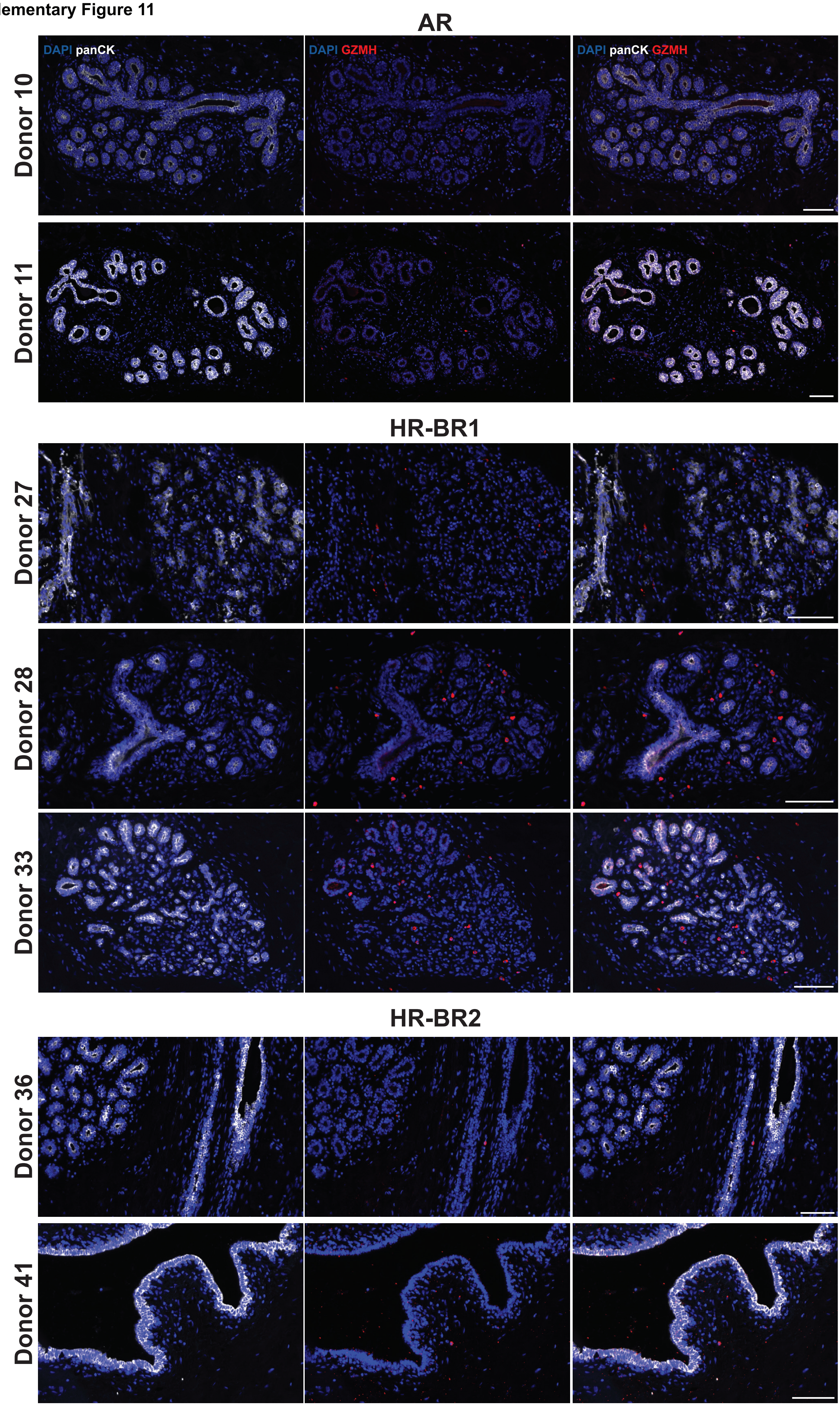

Supplementary Figure 11. GZMH expression in AR and HR donors.

Immunofluorescence staining showing panCK (white), GZMH (red) and DAPI (blue) of representative breast sections from AR and HR donors. Scale bars represent 100 μm.

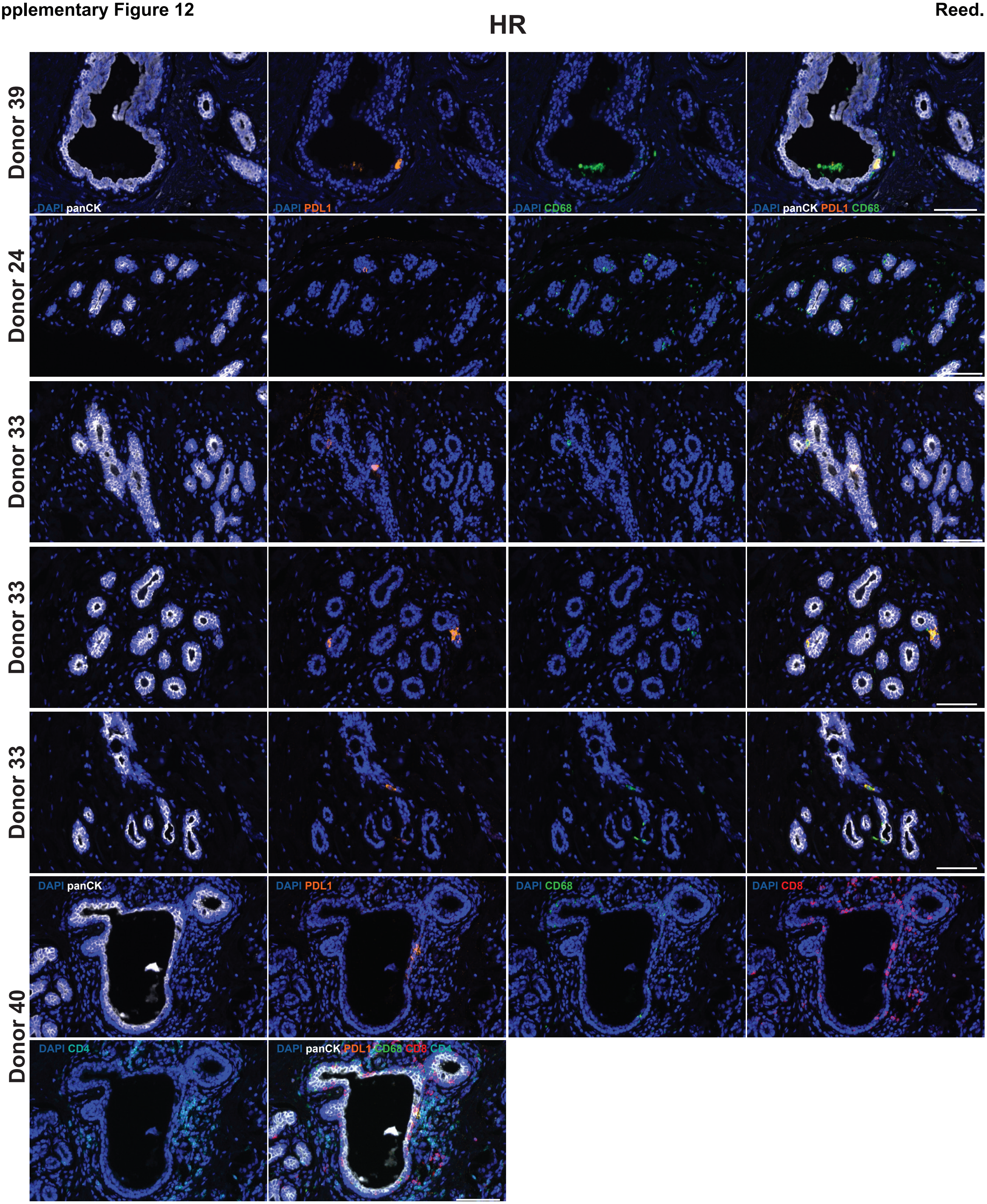

Supplementary Figure 12. PDL1 expression in epithelial and immune cells in HR donors.

Ultivue staining showing panCK (white), PDL1 (orange), CD68 (green) and DAPI (blue) of representative breast sections from HR donors. For donor 40 CD8 (red) and CD4(cyan) are also shown. Scale bars represent 100 μm.

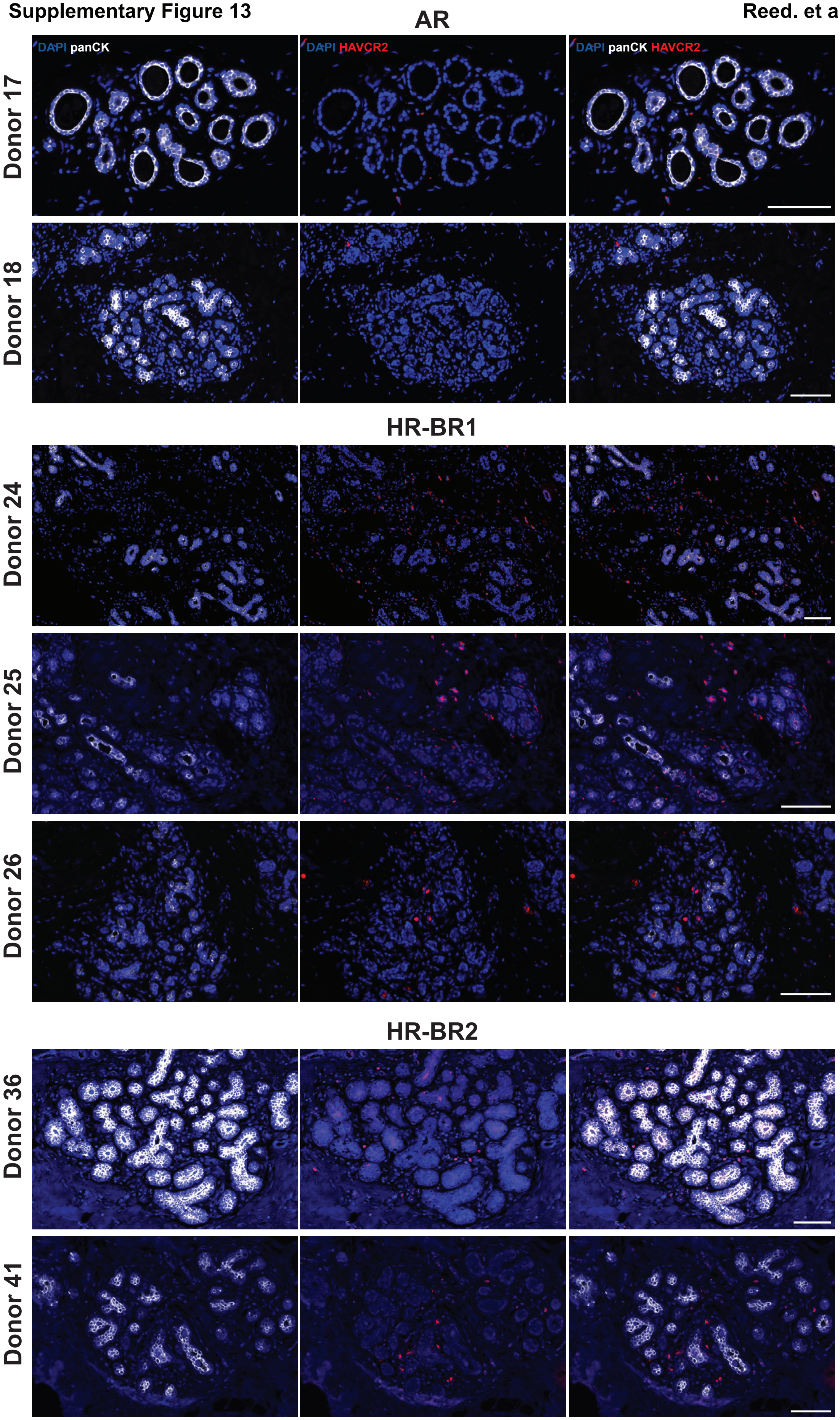

Supplementary Figure 13. HAVCR2 expression in AR and HR donors.

Immunofluorescence staining showing panCK (white), HAVCR2(red) and DAPI (blue) of representative breast sections from AR and HR donors. Scale bars represent 100 µm.

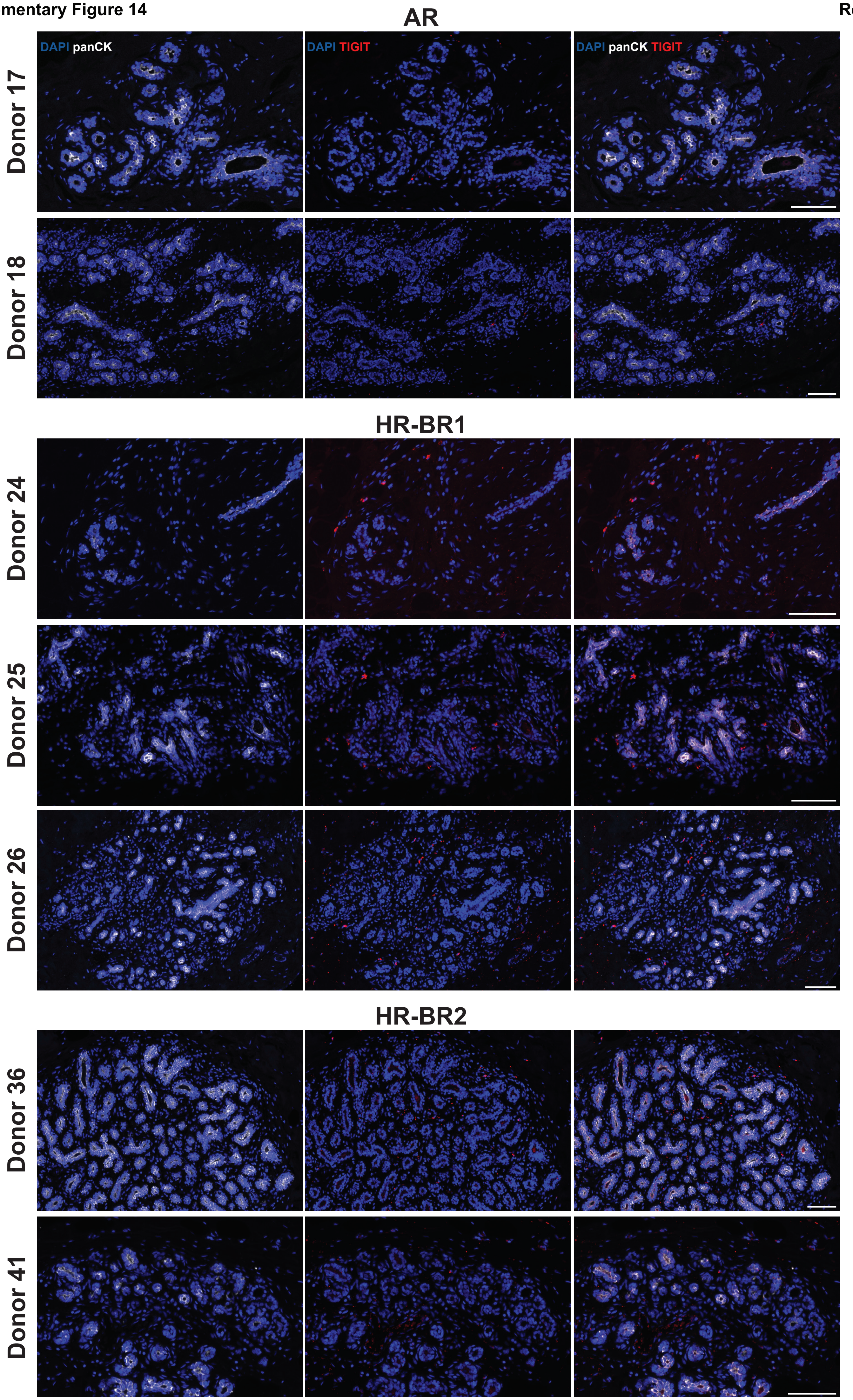

Supplementary Figure 14. TIGIT expression in AR and HR donors.

Immunofluorescence staining showing panCK (white), TIGIT (red) and DAPI (blue) of representative breast sections from AR and HR donors. Scale bars represent 100 μm.

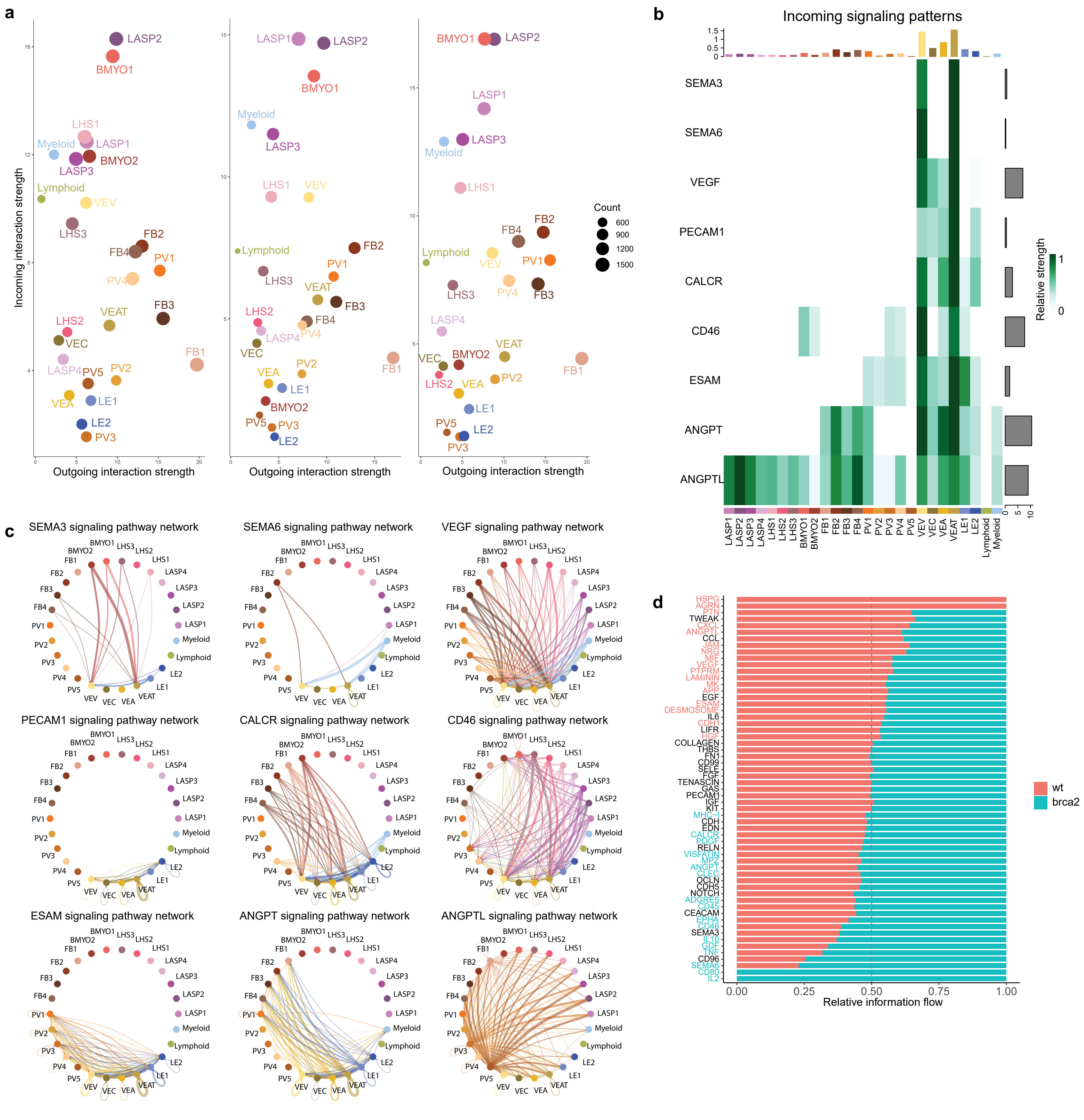

Supplementary Figure 15. Cell-cell communication of high-risk donors

(a) Scatter plot showing the overall changes in total incoming and outgoing cell-cell interactions (through Cell Chat predicted ligand-receptor interactions) comparing average risk (AR) to high-risk BRCA1 germline (HR-BR1) and high-risk BRCA2 germline (HR-BR2) donors.

(b) A heatmap displaying the nine interaction pathways with most specific incoming signal to the endothelial angiogenic tip cells.

(c) Circle plots of the nine most specific incoming signaling pathways to endothelial tip cells. The lines indicating directed cell-cell communication are coloured by the 'sender' cell subcluster and lead towards the receiving cell subcluster and their width is determined by the number of these predicted interactions.

(d) A relative information flow bar plot showing the relative changes in proportions of cell-cell communication pathways in AR and HR-BR2 donors. The pathway axis labels are coloured blue/red if they are significantly ($p < 0.05$) up/downregulated in HR-BR2 donors in a two-sided Wilcoxon test.
