# Supplementary material for: A single-cell atlas enables mapping of homeostatic cellular shifts in the adult human breast
Source: Nat Genet. 2024 Mar 28;56(4):652–62. doi: 10.1038/s41588-024-01688-9 (PMC11018528; doi:10.1038/s41588-024-01688-9)
Supplement: Supplementary file 2 — Reporting Summary [file 41588_2024_1688_MOESM2_ESM.pdf]

nature portfolio

Corresponding author(s): Walid Khaled

Last updated by author(s): 2024/01/22

Reporting Summary

Nature Portfolio wishes to improve the reproducibility of the work that we publish. This form provides structure for consistency and transparency in reporting. For further information on Nature Portfolio policies, see our Editorial Policies and the Editorial Policy Checklist.

Statistics

For all statistical analyses, confirm that the following items are present in the figure legend, table legend, main text, or Methods section.

n/a	Confirmed	
☐	☒	The exact sample size (*n*) for each experimental group/condition, given as a discrete number and unit of measurement
☐	☒	A statement on whether measurements were taken from distinct samples or whether the same sample was measured repeatedly
☐	☒	The statistical test(s) used AND whether they are one- or two-sided<br>*Only common tests should be described solely by name; describe more complex techniques in the Methods section.*
☐	☒	A description of all covariates tested
☐	☒	A description of any assumptions or corrections, such as tests of normality and adjustment for multiple comparisons
☐	☒	A full description of the statistical parameters including central tendency (e.g. means) or other basic estimates (e.g. regression coefficient) AND variation (e.g. standard deviation) or associated estimates of uncertainty (e.g. confidence intervals)
☐	☒	For null hypothesis testing, the test statistic (e.g. *F*, *t*, *r*) with confidence intervals, effect sizes, degrees of freedom and *P* value noted<br>*Give P values as exact values whenever suitable.*
☒	☐	For Bayesian analysis, information on the choice of priors and Markov chain Monte Carlo settings
☒	☐	For hierarchical and complex designs, identification of the appropriate level for tests and full reporting of outcomes
☒	☐	Estimates of effect sizes (e.g. Cohen's *d*, Pearson's *r*), indicating how they were calculated

Our web collection on statistics for biologists contains articles on many of the points above.

Software and code

Policy information about availability of computer code

Data collection	No software was used for data collection
Data analysis	Cell Ranger Single-Cell Software Suite (v6.0.2) was used for demultiplexing, barcode assignment and UMI quantification. All downstream computation analyses were performed in R and Python using standard functions unless otherwise indicated. The specific package and language versions used for each section of analysis are specified in conda yaml files included the HBCA GitHub repository referenced in Code Availability of the text (https://github.com/MarioniLab/hbca). The following packages were used for scRNA-seq data analysis: Cell Ranger Single-Cell Software Suite (v6.0.2), Vireo (v0.5.6), DropletUtils (v1.12.1), Scrublet (v0.2.3), scanpy (v1.8.2), edgeR (v3.36.0), Milo (v1.3.1), Cell Chat (v1.6.0), inferCNV (v. 1.10.0), CellTypist (v0.1.9).<br><br>FlowJo V10 was used to analyse flow cytometry data. Qupath v0.4.3, ImageJ v1.54f and Halo v3.6.4134.137 and HighPlex FL v4.2.14 were used to analyse Ultivue and Immunofluorescence images

For manuscripts utilizing custom algorithms or software that are central to the research but not yet described in published literature, software must be made available to editors and reviewers. We strongly encourage code deposition in a community repository (e.g. GitHub). See the Nature Portfolio guidelines for submitting code & software for further information.

Data

Policy information about availability of data

All manuscripts must include a data availability statement. This statement should provide the following information, where applicable:

- Accession codes, unique identifiers, or web links for publicly available datasets
- A description of any restrictions on data availability
- For clinical datasets or third party data, please ensure that the statement adheres to our policy

The authors declare that all data supporting the findings of this study and unprocessed images are available within the article and its supplementary information files or from the corresponding author upon reasonable request. The raw sequencing data and individual sample processed matrices are available on Array Express E-MTAB-13664. Processed data can also be explored and downloaded at the CellXGene site (https://cellxgene.cziscience.com/collections/cd9a09e2-b440-4887-9163-6f8c684c7ced). The trained CellTypist logistic regression models for label transfer can be found and downloaded from DOI: 10.5281/zenodo.10044650.

Research involving human participants, their data, or biological material

Policy information about studies with human participants or human data. See also policy information about sex, gender (identity/presentation), and sexual orientation and race, ethnicity and racism.

Reporting on sex and gender	The samples included in the study were collected from female individuals only, therefore findings only apply to female individuals and no sex-based analysis was performed. No information was collected on gender, therefore no gender-based analysis could be performed.
Reporting on race, ethnicity, or other socially relevant groupings	Metadata linked to the tissue bank samples was provided by BCN. Ethnicity was reported in Extended Data Figure 1 and Supplementary Table 1 when available, however it was not taken into account in the data analysis as it was only reported for a fraction of the samples.
Population characteristics	All primary human breast tissue was derived from women undergoing reduction mammoplasties with no known genetic history (n = 22) and risk reduction prophylactic mastectomies from women with germline BRCA1 or BRCA2 mutations or other family histories (n = 27) and contralateral mastectomies from BRCA1 carriers that had breast cancer in one breast and had the second breast removed to reduce the risk of further tumours (n = 6). No specific age-range was selected.
Recruitment	Participants were not specifically recruited for this study and are part of bigger cohorts where recruitment was not based on the parameters of interest for this analysis.
Ethics oversight	All primary human breast tissue was obtained from the Breast Cancer Now Tissue bank, as approved by REC (15/EE/0192).

Note that full information on the approval of the study protocol must also be provided in the manuscript.

Field-specific reporting

Please select the one below that is the best fit for your research. If you are not sure, read the appropriate sections before making your selection.

☒ Life sciences ☐ Behavioural & social sciences ☐ Ecological, evolutionary & environmental sciences

For a reference copy of the document with all sections, see nature.com/documents/nr-reporting-summary-flat.pdf

Life sciences study design

All studies must disclose on these points even when the disclosure is negative.

Sample size	Sample size was limited by availability of material.
Data exclusions	One 10X sequencing lane belonging to donor 1016CP was removed in early quality control stages due to the sample reading poor quality control metrics and showing large quantities of debris. This was determined a failed lane of sequencing and thus ignored from further analysis. No further data exclusions were made.
Replication	This was an atlas study looking at 55 donors.
Randomization	We used randomization to group samples for sequencing to minimize possible batch effects (see methods for more details). In statistical testing we blocked for effects of age and parity where possible to minimise confounding (see methods for specific details).
Blinding	Blinding was not relevant for this study as no treatments were provided to the participants.

Reporting for specific materials, systems and methods

We require information from authors about some types of materials, experimental systems and methods used in many studies. Here, indicate whether each material, system or method listed is relevant to your study. If you are not sure if a list item applies to your research, read the appropriate section before selecting a response.

Materials & experimental systems

n/a	Involved in the study
☐	☒ Antibodies
☐	☒ Eukaryotic cell lines
☒	☐ Palaeontology and archaeology
☒	☐ Animals and other organisms
☒	☐ Clinical data
☒	☐ Dual use research of concern
☒	☐ Plants

Methods

n/a	Involved in the study
☒	☐ ChIP-seq
☐	☒ Flow cytometry
☒	☐ MRI-based neuroimaging

Antibodies

Antibodies used	CD45-APC (Biolegend, clone H130,1:100), CD31-APC (Biolegend, clone WM-59, 1:100), EPCAM-AF488 (Biolegend, clone 9C4, 1:50), CD49f-PE/Cy7 (Biolegend, clone GoH3, 1 µg ml−1, 1:200),PanCK (1:1000, Novus Bio NBP2-29429), HAVCR2 (1:150, Abcam ab241332), TIGIT (1:200, Abcam ab243903), GZMH (1:200, Atlas Antibodies HPA029200), goat anti-rat AlexaFluor 488, anti-mouse AlexaFluor 568 (1:200, Invitrogen).
Validation	Antibodies were used according to manufacturer specification and validated by manufacturers in house. Immunofluorescence antibodies were validated based on staining pattern and cellular morphology only. Flow cytometry antibodies have been extensively validated in the Khaled lab and all the clones used are extensively implemented in the mammary gland biology field.

Eukaryotic cell lines

Policy information about cell lines and Sex and Gender in Research

Cell line source(s)	HuMECs, Thermo Fisher Scientific A10565
Authentication	No authentication was undertaken.
Mycoplasma contamination	The cell line was not tested for mycoplasma. cells were used as spike-in controls for 10X preps
Commonly misidentified lines (See ICLAC register)	no commonly misidentified cell lines were used in the study

Flow Cytometry

Plots

Confirm that:

☒ The axis labels state the marker and fluorochrome used (e.g. CD4-FITC).

☒ The axis scales are clearly visible. Include numbers along axes only for bottom left plot of group (a 'group' is an analysis of identical markers).

☒ All plots are contour plots with outliers or pseudocolor plots.

☒ A numerical value for number of cells or percentage (with statistics) is provided.

Methodology

Sample preparation	Frozen vials of epithelial-enriched or stromal-enriched fractions were defrosted by gently diluting the material in 50 mL of cold Tissue Preparation Medium (TPM, RPMI 1640 + 25 mM HEPES and 2 mM L-glutamine (Sigma R5886), 5% foetal bovine serum (FBS, Gibco), 100 units/mL penicillin and 0,1 mg/mL streptomycin sulphate (Gibco), washed in PBS without Calcium and Magnesium (D8537, Sigma). Samples were centrifuged at 400 g for 5 minutes and resuspended in 2 mL of freshly prepared PBS + 0,025% Trypsin, 0,1 g/L EDTA (HyClone SV30031.01, Fisher Scientific) and 0,4 mg/mL Deoxyribonuclease 1 (DNase) (10104159001, Boehringer/Roche Diagnostics) previously warmed to 37°C. Samples were then incubated at 37°C with pipetting up and down for 30 seconds every 2-3 minutes until smoothly digested or up to a maximum of 10 minutes. Next, samples were washed in 40 mL of TPM and centrifuged for 20 minutes at 400 g with slow break. The pellet was resuspended by pipetting up and down in 200 µL of TPM +10 µL of 10 mg/mL DNase until homogeneous, then diluted in 25 mL of TPM and filtered through a 40 µm cell strainer (352354, Corning) into a 50 mL tube. After centrifugation for 5 minutes at 400 g, the pellet was resuspended by pipetting up and down in 200 µL of CPM (Cell Preparation Medium, RPMI 1640 + 1% FBS, 100 units/mL penicillin, 0,1 mg/mL streptomycin sulphate) + 10 µL of 10 mg/mL DNase until homogeneous, then washed in 3-6 mL of CPM. 30,000 cells were resuspended into 48 µL of HF (Hank's balanced salt solution (Gibco)+1% FBS) into low binding tubes. 400 Human mammary epithelial cells (HuMECs, Thermo Fisher Scientific A10565) were added as spike-in, and samples were submitted for scRNAseq (unsorted fraction). For the epithelial-enriched fraction only, the rest of the processed

	sample was stained with the following primary antibodies: CD45-APC (Biolegend, clone H130,1:100, staining most hemopoietic cells), CD31-APC (Biolegend, clone WM-59, 1:100, staining endothelial cells), EPCAM-AF488 (Biolegend, clone 9C4, 1:50), CD49f-PE/Cy7 (Biolegend, clone GoH3, 1 µg ml−1, 1:200). DAPI was used to detect dead cells. Cells were filtered through a cell strainer (Partec) before sorting. Sorting of cells was done using a FACS Aria Fusion sorter. Single-stained control cells were used to perform compensation manually and unstained cells were used to set gates. After doublets, dead cells and contaminating haematopoietic and endothelial cells (referred to as lineage) were gated out (Supplementary Fig 2), up to 30,000 luminal progenitors were sorted for scRNAseq (with the addition of 400 HuMECs as spike-in).
Instrument	FACS Aria Fusion
Software	FlowJo
Cell population abundance	Purity of samples was not determined post-sorting.
Gating strategy	After doublets, dead cells and contaminating haematopoietic and endothelial cells (referred to as lineage) were gated out, EPCAM+, CD49F+ luminal progenitors cells were sorted. The gating strategy is reported in Supplementary Fig 2. Starting cell population values for FSC include all cells between 25k and 250k, and the whole range of SSC was included.

☒ Tick this box to confirm that a figure exemplifying the gating strategy is provided in the Supplementary Information.
